# Stem Cell Promise, Interrupted: How Long Do US Researchers Have to Wait?

**DOI:** 10.1371/journal.pbio.0050032

**Published:** 2007-01-16

**Authors:** Liza Gross

The summer of 2006 was a heady time for neurologist Douglas Kerr. As Director of the Johns Hopkins Transverse Myelitis Center in Maryland, Kerr studies the mechanisms of neurodegenerative diseases in the hope of developing therapies to treat them. He sees patients with transverse myelitis, amyotrophic lateral sclerosis, and spinal muscular atrophy (SMA), an inherited disorder that in its most severe form renders newborns limp and “floppy,” unable to suck, swallow, or breathe. Kerr's voice tightens as he describes the fate of these babies, many of whom will die before their second birthday. He's convinced that embryonic stem cells will one day help people with progressive, motor-neuron-destroying disorders recover control of their movements, and their lives.

For six long years, Kerr's team pursued the elusive elixir that would restore mobility to the paralyzed adult rats he uses to model neurodegeneration in humans. The researchers had cleared two major technical hurdles early on: they managed to derive spinal motor neurons from mouse embryonic stem cells in sufficient numbers to transplant in the rats' spinal cords, and they ensured the transplanted neurons' survival. But they struggled for years to prod the spinal motor neurons to send their axons out of the spinal cord and form functional neuromuscular junctions with the lame muscle.

Finally, in 2005, they hit the mark. Growth factors injected into the spinal cord induced the transplanted motor neurons to form connections with resident neurons. A second set of growth factors overcame inhibitors in myelin (the protective sheath around nerves that blocks axon growth in adult animals), allowing the motor neurons to send their axons out of the spinal cord toward skeletal muscle. And yet another growth factor injected into the muscle stimulated functional connections between the neurons and muscle. Kerr watched his rats—immobilized with a motor-neuron-destroying virus—move hind limbs that had been paralyzed for nearly four months. (Watch before and after videos of the rats on the Johns Hopkins Web site, http://www.hopkinsmedicine.org/Press_releases/2006/Mousevideo.html.)

When Kerr and his colleagues reported their results in the June 2006 online version of *Annals of Neurology*, the work was widely hailed as the first evidence that stem-cell-based therapy could recapitulate early developmental signals and rewire a damaged neural circuit. Elias Zerhouni, Director of the US National Institutes of Health (NIH), which funded part of the research, called the work a “remarkable advance” demonstrating the power of stem cells to treat neurodegenerative diseases. All those years of frustration had finally paid off. But would the technique work in humans?

To find out, Kerr must use motor neurons derived from human embryonic stem cells (hESCs) and show that they can establish functional connections with skeletal muscle over the longer distances found in a larger animal. (He's settled on pigs.) He must also show that the treatments are safe. If the pig experiments generate the necessary safety and efficacy data, he will submit his results to the US Food and Drug Administration, seeking approval for a clinical trial to use the hESC-derived motor neurons in babies with fatal SMA.

**Figure:**
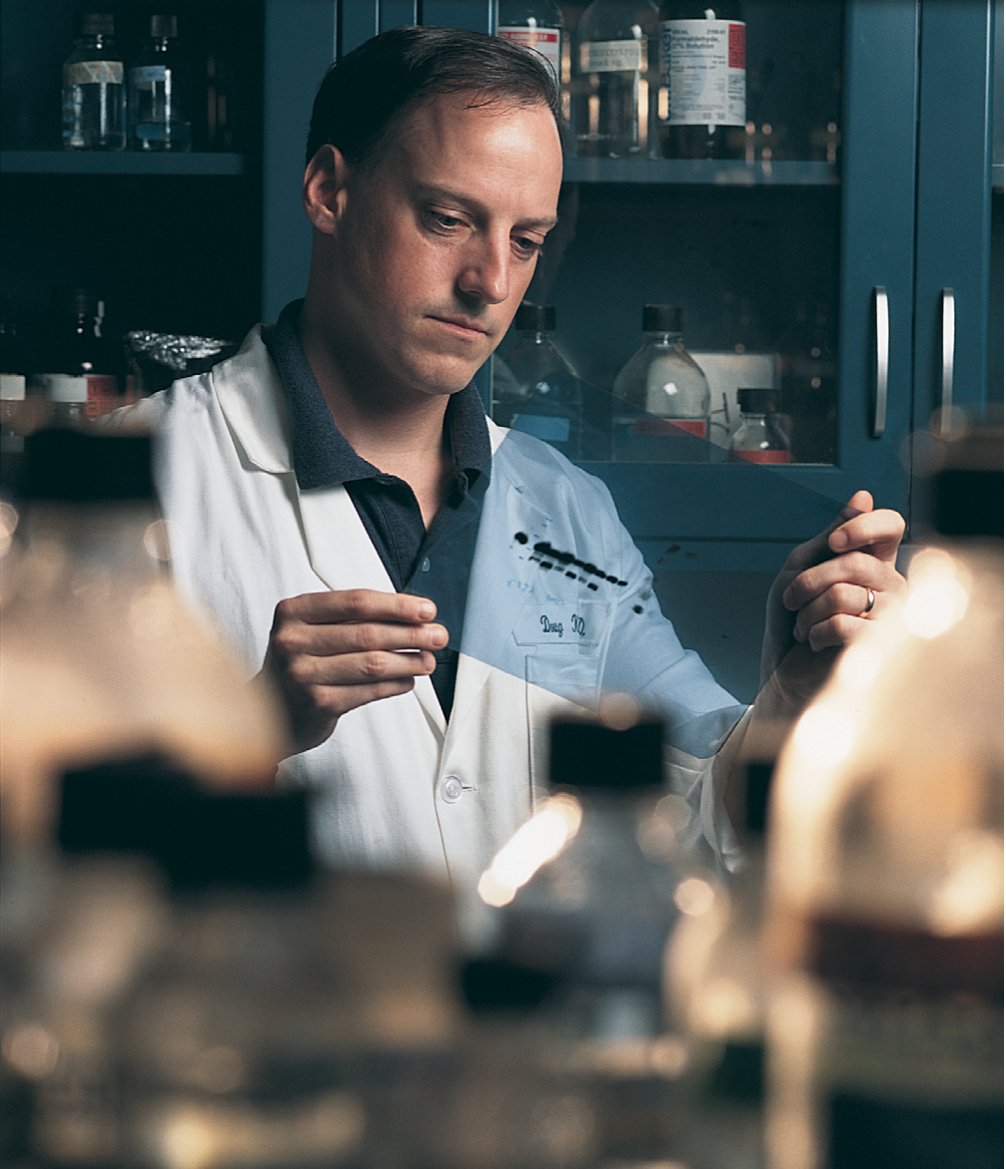
Douglas Kerr explores the promise of embryonic stem cells to treat neurodegenerative disorders

Kerr chose babies with SMA for the first clinical trials, he explains, because infants have less myelin to inhibit axon growth, so the chance of re-innervation is greater. Their neurons need travel just a short distance compared to adults, and the developmental cues that guide axon growth toward their appropriate targets are still in place. And because no treatment or cure exists for these babies, an experimental treatment represents their only hope. Kerr had planned to use federally approved hESCs until he found out that the federal lines could not reliably yield motor neurons with anywhere near the efficiency of newer lines generated with private funds. (In the rat experiments, each animal had 60,000 motor neurons transplanted into their spinal cord.)

Still, Kerr watched hopefully as a bipartisan bill authorizing expanded federal financing of hESC research passed the US House of Representatives in 2005 and then the Senate last year. In addition to allowing federally funded researchers to derive new hESC lines from embryos awaiting destruction in fertility clinics, the Stem Cell Research Enhancement Act would lift the ban on lines derived after August 2001. But President George W. Bush vetoed the bill in July 2006, and “put a real chill on things,” Kerr says. Now Kerr is worried that if he acquires the pigs and prepares them for the stem cell therapy, he'll run into a brick wall when the time comes to get the hESCs he needs for the transplantation experiments.

Even though Maryland passed a measure in 2006 to spend $15 million on hESC research, Kerr says that's just a one-time appropriation. Working with live animals costs several million dollars. “What am I going to do next year when I've got all those animals?” he asks.

Kerr won't qualify for federal funding if he uses non-approved lines, but he's not willing to risk the potential problems with the federally sanctioned lines. And adult stem cells aren't an option. He tried to generate spinal motor neurons from adult stem cells and cells isolated from umbilical cord blood, but decided that programming a blank slate—hESCs—is far more efficient than deprogramming specialized cells and redirecting them toward a different fate. He put everything on hold, pending the outcome of the November midterm elections.

## A Political Straitjacket

The use of federal funds to create or destroy human embryos for research was outlawed in the US by Congress in 1997. On August 9, 2001, when President Bush announced his decision to allow federal funding to support hESC research only on lines already derived—because “the life and death decision has already been made”—scientists were just learning what the cells needed to thrive and maintain their “stemness,” the ability to self-renew and differentiate into any cell type of the body (called pluripotency). Methods used to derive these early hESC lines were technically demanding and labor-intensive, requiring the artful touch of a highly skilled technician to tease apart cells with a glass needle to propagate the cell lines (a technique called mechanical passaging). The lines were also grown on mouse fibroblasts—cells that act as “feeder” layers to maintain the stem cells in an undifferentiated state—increasing the risk that the human cells would absorb mouse molecules and trigger rejection by the immune system if used in clinical trials.

Scientists have since figured out how to wean the human cells off the mouse feeder cells, but the process is time-consuming. A team of researchers working in Miodrag Stojkovic's lab at the University of Newcastle, UK, developed a method of deriving hESCs that eliminates the risk of contamination from both mice and human donors. In their “autogenic” feeder system, a parental hESC line gives rise to a subset of differentiated fibroblasts that sustain the parental line. While Stojkovic's lines were grown on medium containing animal products, he says that this approach demonstrates how researchers can generate clinical-grade hESC lines that would meet the Federal Drug Administration's Good Manufacturing Practices safety requirements. Last July, the Singapore biotech company ESI announced that it had derived four safe lines of hESCs for clinical use. And the Hadassah Medical Organization in Israel recently developed animal-free methods for isolating pluripotent stem cells from human embryos (obtained from in vitro fertilization clinics) and deriving new clinical-grade hESC lines. These are just the types of cells Kerr could use for his pig experiments—if he were free to use any lines he wanted.

Kerr had planned to use federally approved hESCs until he found out that the federal lines could not reliably yield motor neurons with anywhere near the efficiency of newer lines generated with private funds.

Carol Ware, Director of the Human Embryonic Stem Cell Core Laboratory at the University of Washington School of Medicine, has been working to characterize the available NIH-approved hESC lines. So far, Ware and her colleagues have tested 14 of the 22 available lines (a 15th line arrived at the lab contaminated with mycoplasma) for growth efficiency, genomic stability, appropriate gene expression during self-renewal and differentiation, and other NIH criteria.

The team found considerable variation among the lines. Some lines had a propensity to develop chromosomal abnormalities over time, and others were hard to grow. “Certain lines are very difficult to thaw,” Ware says, “so you may only get one or two cells.” Not a great return for cell lines that cost between $2,500 and $6,000.

Complicating matters further, the cells seem to prefer the culture conditions in which they were derived. All of the lines were originally derived through mechanical passage. And though some were eventually converted to enzymatic passage—a speedy, less onerous technique that has become the standard method for expanding hESC lines—it's not possible to predict which lines can convert to enzymatic passage, further compromising their utility.

Mechanically passaging lines is “a real pain in the neck,” says Larry Goldstein, Director of the University of San Diego Stem Cell Program, likening the technique to early versions of a software program that still need debugging. Goldstein's lab has been exploring the properties of some of the federal lines, and still uses one of the approved lines in experiments. “It's been okay in some areas, and a little tougher in others,” he says. “Others use it and are happy with it.”

The lines “certainly aren't useless,” he adds, but he's found them clumsy to handle. “For our experiments, we need cell lines that grow well for more than just the most-skilled tissue person. If only one person in the lab is sufficiently skilled to grow them, you're not going to get much done.” Goldstein plans to investigate non-approved lines with private or state funding.

Ware found that lines also varied in their ability to form different tissues, suggesting that each line may possess unique capabilities. This variable behavior may arise from differences in the way the lines were derived or in the inherent properties of the cells themselves, the team reported online on August 17, 2006, in *Stem Cells*. Either way, they concluded, it underscores the need to derive and study additional hESC lines.

Harvard researcher Doug Melton came to the same conclusion even before the Bush administration restrictions were put in place, when he began looking for hESCs for his work on type I diabetes in the late 1990s. Disappointed with what he found, he decided to generate his own lines, and in 2004, Melton and his team announced that they had derived 17 new hESC lines, using funding from the Howard Hughes Medical Institute and the Juvenile Diabetes Research Foundation. Melton's team used enzymatic passaging to make the lines more user-friendly, allowing far more labs to handle the cells, provided they find state or private funding. The Harvard lines, as they're known, are also available free of charge.

Ware believes the federal lines will become historical lines, as technological advances have already made the older lines seem outdated. “We're understanding culture techniques so much better now, and as you understand more and more what the cells want, you're going to get better lines.”

## States and Private Donors Step In

US advocates of stem cell research read the 2006 midterm election results as a sign that embryonic stem cell research has gained widespread bipartisan support. They point to Missouri as the bellwether state. For the past five years, Missouri lawmakers tried to pass a measure to criminalize stem cell research in the state. But last November, voters not only approved a state constitutional amendment protecting stem cell research, but ousted incumbent US Senator Jim Talent, who called stem cell research “morally reprehensible,” in favor of Claire McCaskill, a vocal supporter of hESC research. Stem cell research figured prominently in six US Senate races; in each case, the candidate who supported stem cells won.

“The political dynamic in the 110th Congress is going to be a lot different than it was in the 109th,” says Sean Tipton, President of the Coalition for the Advancement of Medical Research, a stem cell advocacy group. Tipton thinks the new Congress will be even more supportive than the 109th, which passed the Stem Cell Research Enhancement Act with strong bipartisan support.

Douglas Kerr goes even further. He's confident the bill will pass this year with a veto-proof majority, and that “we'll have federal funding in 2007.”

But what the next year holds is unclear. Nancy Pelosi, who will preside over the House of Representatives as Speaker when the new Congress convenes in January, has pledged to “promote stem cell research to offer real hope to the millions of American families who suffer from devastating diseases” in Congress's first 100 hours. But an analysis based on the stated stem cell positions of the newly constituted House and Senate by *The Chronicle of Higher Education* found that if the stem cell act were reintroduced, votes in the House would fall short of a veto-proof majority. And few doubt that Bush would exercise his veto prerogative.

While the federal prospects remain uncertain, states are increasingly filling the void. A 2006 Congressional Research Service report to Congress lists 12 states as actively encouraging or funding stem cell research, with Wisconsin and California leading the way. The California Institute of Regenerative Medicine (CIRM), created to oversee the $3 billion stem cell research program authorized by voters in 2004, awarded $12 million in training grants last April and expects to award over 55 research grants totaling over $100 million in early 2007. Grant allocations were initially stalled by lawsuits filed by pro-life and anti-tax groups, until Governor Arnold Schwarzenegger stepped in with a $150 million state loan, and private donors and foundations pledged $45 million in loans against the bond to get the ball rolling.

All this support from states and private donors puts more scientists to work, says Dale Carlson, Chief Communications Officer for CIRM. With the uncertainty at the federal level, he says, it's important that the states and private donors are stepping in, “instead of scientists stepping back and waiting till the policy changes.”

While senior scientists acknowledge the difficulty of recruiting the best young minds to a field so mired in controversy, those hot on the trail of potential cures using stem cells are not about to sit idly by while Washington fiddles. Kerr is hoping for the best in 2007, but he and his California collaborator, Hans Keirstead, are pursuing nonfederal funding “while we await changes in D. C.” Both are appealing to private philanthropy groups for bridge funding to make sure their work continues.

Ware and her colleagues have tested 14 of the 22 available NIH lines for growth efficiency, genomic stability, appropriate gene expression during self-renewal and differentiation, and other NIH criteria, and found considerable variation among the lines.

In November, CIRM received 70 applications for comprehensive research grants totaling $80 million. University of California San Diego's Goldstein was among the applicants, voicing frustration with the limitations on scientific freedom imposed by the federal restrictions. “Bush's policy hasn't spared any frozen embryos as far as I know,” he says. “The biggest destruction of human blastocysts happens in IVF clinics and that hasn't changed.”

Meanwhile the research moves ahead without the centralized control and oversight of the federal government. The lack of federal support means that US researchers—who led the way in setting standards for genetic testing and genome sequencing—cannot do the same for embryonic stem cell research. Although CIRM and the National Academy of Sciences are setting research guidelines for state-funded researchers, if US researchers aren't at the forefront of the field, they can't lead by example.

But scarce research dollars, some researchers believe, is an even bigger problem. In an era of shrinking NIH budgets and heightened competition for federal grants, restricting grantees to the less tractable NIH-approved lines means the federal government is spending less money on stem cell research, and spending it less efficiently. “As a scientist you want to have the maximum number of tools available because the research problems are hard enough, even when the lines behave well,” Goldstein says. “And these aren't just theoretical problems. We're trying to figure out what's gone wrong with these terrible diseases that afflict large numbers of people.” The federal restrictions, he says, have forced the community to work with one hand tied behind its back.

US advocates of stem cell research read the 2006 midterm election results as a sign that embryonic stem cell research has gained widespread bipartisan support.

That means that if a US researcher who has access to ten or 15 federally approved hESC lines is working on the same question as a researcher in Singapore, for example, who may have access to 100 lines, the US researcher can't hope to compete. With reports that some lines appear genetically predisposed to behave one way or another, therapeutic applications may require creating stem cell lines that are genetically identical to the patient, to prevent immune rejection. That's a question that researchers like Douglas Kerr can't ask if they're restricted to the limited diversity of ten hard-to-grow stem cell lines.

With the right cells in hand, Kerr would first seek proof of principle that his neuroregenerative stem cell therapy can work in pigs, and then move on to see if it can help babies with SMA—a path he's charting in a grant right now. If the results showed that the researchers were on the right track, they would move on to test this type of therapy in patients with the more complex lesions found in transverse myelitis, amyotrophic lateral sclerosis, and traumatic spinal cord injury. Aside from a political sea change that lifts the federal restriction on stem cell research—a shift that may have arrived with the 110th Congress, or may still come up a few votes short—what does Kerr's group need to move forward? “If we got ten good lines that were genetically normal, and had not come into contact with other species, and could become motor neurons,” he says, “we'd be set.”

## Abbreviations

CIRM: California Institute of Regenerative Medicine
hESC: human embryonic stem cell
NIH: National Institutes of Health
SMA: spinal muscular atrophy
